# Design and Optimization of a Nanoparticulate Pore Former as a Multifunctional Coating Excipient for pH Transition-Independent Controlled Release of Weakly Basic Drugs for Oral Drug Delivery

**DOI:** 10.3390/pharmaceutics15020547

**Published:** 2023-02-07

**Authors:** Hao Han R. Chang, Kuan Chen, Jamie Anne Lugtu-Pe, Nour AL-Mousawi, Xuning Zhang, Daniel Bar-Shalom, Anil Kane, Xiao Yu Wu

**Affiliations:** 1 Advanced Pharmaceutics and Drug Delivery Laboratory, Leslie Dan Faculty of Pharmacy, University of Toronto, Toronto, ON M5S 3M2, Canada; 2 Department of Pharmacy, University of Copenhagen, 2100 Copenhagen Ø, Denmark; 3 Patheon by Thermo Fisher Scientific, Toronto Region Operations (TRO), Mississauga, ON L5N 3X4, Canada

**Keywords:** multifunctional terpolymer excipient, controlled release, microenvironmental pH modifier, pore former, pH-responsive nanoparticles, nanogel optimization, weakly basic drug, bilayer-coated beads, pH-independent release

## Abstract

Bioavailability of weakly basic drugs may be disrupted by dramatic pH changes or unexpected pH alterations in the gastrointestinal tract. Conventional organic acids or enteric coating polymers cannot address this problem adequately because they leach out or dissolve prematurely, especially during controlled release applications. Thus, a non-leachable, multifunctional terpolymer nanoparticle (TPN) made of cross-linked poly(methacrylic acid) (PMAA)-polysorbate 80-grafted-starch (PMAA-PS 80-g-St) was proposed to provide pH transition-independent release of a weakly basic drug, verapamil HCl (VER), by a rationally designed bilayer-coated controlled release bead formulation. The pH-responsive PMAA and cross-linker content in the TPN was first optimized to achieve the largest possible increase in medium uptake alongside the smallest decrease in drug release rate at pH 6.8, relative to pH 1.2. Such TPNs maintained an acidic microenvironmental pH (pH_m_) when loaded in ethylcellulose (EC) films, as measured using pH-indicating dyes. Further studies of formulations revealed that with the 1:2 VER:TPN ratio and 19% coating weight gain, bilayer-coated beads maintained a constant release rate over the pH transition and exhibited extended release up to 18 h. These results demonstrated that the multifunctional TPN as a pH_m_ modifier and pH-dependent pore former could overcome the severe pH-dependent solubility of weakly basic drugs.

## 1. Introduction

Many existing active pharmaceutical ingredients (APIs) (drug compounds) are either weak acids or weak bases; their water solubility can change significantly with the lumen pH changes along the gastrointestinal tract (GIT) as a result of variation in the ionization degree. Severe pH-dependent solubility could pose a great challenge to achieving consistent and predictable performance of oral dosage forms, as abrupt changes in release rate may result in unexpected dissolution, absorption, and bioavailability of the drug, leading to increased risks of adverse side effects or decreased therapeutic efficacy. This problem may be pronounced for weakly basic drugs with low solubility at high pH, especially those with a narrow therapeutic index, or requiring prolonged release, because in the lower GIT the pH is >6.8 [1]. Furthermore, food intake, disease state (e.g., inflammatory bowel disease, gastritis, colitis), concomitant medication (e.g., proton pump inhibitors), and inter- and intra-individual variations, among other factors, can alter the pH in the GIT, which deviates from the pH of simulated gastric and intestinal fluid for in vitro testing and prediction [2,3,4]. Hence, novel strategies to formulate weakly basic drugs with extreme pH-dependent solubility in controlled release forms could enhance the repertoire of advanced medications available to patients.

To compensate for the varying pH values in the GIT, several approaches have been employed. One approach is the addition of small-molecule pH modifiers to the immediate vicinity of the drug to change the microenvironmental pH (pH_m_), thus enhancing the drug solubility. For example, organic acids (e.g., adipic, fumaric, and succinic acids) have been introduced into formulations of weakly basic drugs, reducing the pH_m_ sufficiently, thereby facilitating drug dissolution, irrespective of the pH of the bulk solvent [5,6,7,8,9,10,11]. Drug release will depend on the compatibility of the organic acid with the drug in terms of the buffering capacity of organic acids and the pK_a_ of the drug. Nevertheless, the incorporation of organic acids remains challenging as they are prone to leach out from the formulation, leading to inefficient pH modulation over time [12]. Therefore, large amounts of organic acids are required in order to achieve prolonged pH-independent drug release [5,13], which often makes this approach inadequate for controlled release formulations.

Another approach to compensate for the reduction in drug solubility is the use of enteric coating polymers on tablets, pellets, or beads as permeability modifiers, which can increase drug permeability at higher pH. In such coatings, polymers with pH-dependent solubility or swellability are employed, either alone or incorporated in a hydrophobic polymer (e.g., ethylcellulose (EC)) that acts as a membrane barrier to drug diffusion, resulting in slowed drug release. For example, methacrylic acid–ethyl acrylate copolymer (Eudragit^®^ L) and hydroxypropyl methylcellulose acetate succinate are frequently incorporated into these membranes as pore forming materials for pH-dependent release [13,14,15,16,17,18,19,20,21,22,23]. At a pH above their pKa’s, the pore formers dissolve and leach out of the membrane film to form channels that facilitate drug diffusion. However, as pore formers leach out, the film becomes more porous and weaker, increasing the risk of dose dumping due to weakened structure or ruptures of the coating [24].

Recent advances using biopolymer-based nanomaterials, molecular imprinted polymers, and mathematical and computational models have been used to address the various challenges of drug delivery systems [25,26,27,28,29]. Biopolymers such as starch, collagen, chitosan, etc., are useful for their biocompatibility, biodegradability, and ease of synthesis and modifications [25]. Controlled release dosage forms capable of being flexible, releasing drugs in a timely manner, with desired duration and dosage, are more important than ever, and mathematical and computational models also function as influential tools in addressing the potential mechanisms or impediments of drug delivery systems [26,27,28,29].

Previously, a crosslinked terpolymer nanoparticle (TPN), consisting of poly(methacrylic acid)-polysorbate 80-grafted-starch (PMAA-PS 80-g-St) [30,31,32,33], was incorporated into ethylcellulose films (TPN-EC) to respectively reduce or enhance the permeability of the film coating by a pH-dependent shrinking (at low pH) and swelling (at high pH) mechanism [34]. Unlike other soluble polymeric pore-formers, the TPN did not increase the viscosity of EC dispersions for coating, attributable to its crosslinking structure [31,32,33]. For the same reason, TPN did not leach out from cast TPN-EC films, maintaining good mechanical properties compared to conventional Eudragit^®^ L-EC films. When used as a membrane coating over beads loaded with a water-soluble drug, diltiazem HCl, TPN-EC provided faster drug release at pH 6.8 than at pH 1.2 [34]. The biocompatibility of TPN was demonstrated previously in vitro using isolated rat hepatocytes [32].

Inspired by previous findings regarding the pH-dependent properties of TPN, in this work, we explore the application of TPN for the first time to develop an advanced, controlled release bilayer-coated bead formulation for weakly basic drugs that exhibits severe pH-dependent water solubility. Verapamil HCl (VER) was selected as a model drug because it undergoes extreme decrease in solubility by several orders of magnitude when the media pH is increased from acidic to neutral and weakly basic conditions [9,35]. By exploiting the pH-dependence of TPN, we proposed that an increase in permeability at high pH could help compensate for the low solubility of the drug in its unionized form, thereby permitting a constant release rate when transitioning from gastric to intestinal pH. Additionally, we explored the ability of TPN to serve as a pH_m_ modifier, owing to the presence of its acidic functional groups in MAA. Because TPN is retained within EC, unlike traditional leachable pH_m_ modifiers when formulated together with the drug in a matrix, the source of the acidifying agent could be sustained throughout dissolution, while preserving dosage form integrity.

**Figure 1:**
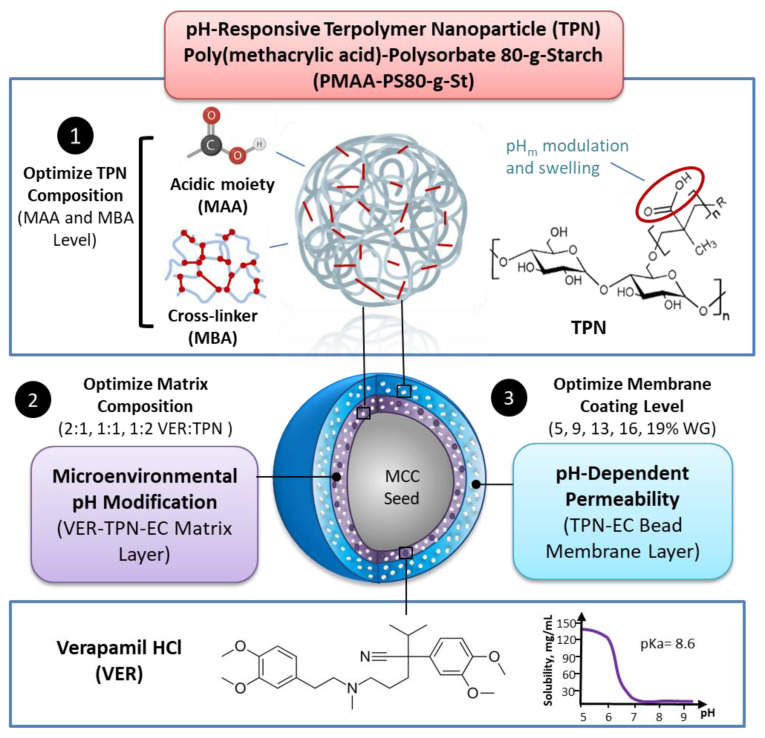
A flow chart of the optimization strategy to achieve pH transition-independent controlled release of VER from a TPN-containing bilayer-coated beads. Formulation strategy for TPN bilayer-coated beads containing weakly basic VER Optimization of TPN composition to achieve pH_m_ modification and pH-dependent swelling, followed by strategic placement of TPN in the bilayer bead matrix and membrane layers are proposed to overcome pH-dependent solubility of VER. Figure created with BioRender.com (accessed on 15 December 2022).

To investigate the effectiveness of combining both the permeability and the pH_m_ modulation strategies of TPN, experiments were performed using EC matrix free-films incorporated with VER and TPN (VER-TPN-EC) and a bilayer-coated bead design consisting of an inner VER-TPN-EC matrix, surrounded by an outer membrane composed of TPN-EC. As illustrated in Figure 1, the TPN composition was first adjusted by varying the amounts of the pH-responsive monomer, MAA, and cross-linker, N,N′-methylenebis(acrylamide) (MBA) for pH-dependent swelling (medium uptake) and drug release via experiments using composite free-films. The best-performing TPN-containing films were then tested for their ability to lower pH_m_. Subsequently, the TPN was incorporated into a bilayer-coated bead design, where it was expected to lower the pH_m_ in the matrix layer and regulate permeability in the membrane layer by its pH-dependent swelling. The effects of formulation parameters such as VER:TPN ratio within the matrix layer and membrane coating level on the pH-independence of dissolution rate in various pH conditions were evaluated.

Our cumulative research investigating the various applications of TPN (e.g., nanoparticle drug carrier in injectables, enteric coating agent and pore former in film coatings, recrystallization inhibitor in amorphous solid dispersions) is helping us widen the breadth of its capabilities. Namely, in the present research, the ability of TPN for pH_m_ modification, pH-responsive swelling, nanoscale pore formation, interaction with drugs, and non-leachability comprise a set of multi-faceted features that set it apart from traditional excipients, which could improve the efficacy and quality of controlled release dosage forms for weakly basic drugs. As the landscape of new drug molecules continues to shift towards greater challenges (e.g., poor solubility, pH-dependent solubility, narrow therapeutic index, etc.) the need for more advanced, multifunctional excipients can be expected to increase.

## 2. Materials and Methods

### 2.1. Materials

Soluble corn starch, methacrylic acid (MAA), N,N′-methylenebisacrylamide (MBA), sodium thiosulfate (STS), potassium persulfate (KPS), sodium dodecyl sulfate (SDS), and sodium phosphate tribasic were purchased from Sigma Aldrich (Oakville, ON, Canada). Verapamil HCl (VER) was purchased from Spectrum Chemicals, (New Brunswick, NJ, USA). Hydrochloric acid (HCl) was purchased from Caledon (Georgetown, ON, Canada). SNARF-4F (#S23920) was purchased from Fisher Scientific (Ottawa, ON, Canada). Bromocresol green was purchased from Sigma Aldrich (Oakville, ON, Canada). Ethylcellulose (Surelease^®^ E-7-19040) was kindly donated by Colorcon (West Point, PA, USA). Polyvinylpyrrolidone (PVP) (Kollidon^®^/PVPK30) was kindly donated by BASF (Ludwigshaven, Germany). Polysorbate 80 (Tween 80-LQ-(CQ)) was kindly donated by Croda (Edison, NJ, USA). Microcrystalline cellulose (MCC) beads (VIVAPUR^®^ MCC Spheres 700) were used as the coating substrate (purchased from JRS Pharma, Weissenborn, Germany).

### 2.2. Synthesis and Characterization of TPN

In an attempt to determine the optimal composition of TPN for pH modulation, the amounts of ionizable monomer (MAA) and cross-linker (MBA) were varied according to the formulations listed in Table 1. TPNs were synthesized by a one-pot aqueous-based free radical polymerization using a KPS/STS redox initiator system in a 250 mL two-necked flask connected to a condenser immersed in a water bath above a magnetic stirrer [33,34]. Corn starch (4.9 mmol) was first dissolved in 180 mL of distilled deionized water (DDIW) at 95 °C for 30 min. The starch solution was then cooled to 65 °C and purged with nitrogen for 30 min to remove dissolved oxygen. Then, 0.45 mmol of KPS and 1.36 mmol of STS were added to the flask and stirred for 10 min. Next, 0.69 mmol of SDS dissolved with PS 80 in 10 mL of DDIW was added and stirred for 5 min. Finally, MAA and MBA, dissolved in 10 mL of DDIW, were added to the flask to start the reaction. The reaction was left overnight at 65 °C to guarantee complete polymerization. Subsequently, the product was neutralized to pH 7 with 2 N NaOH. The product was then purified by centrifugation twice at 96,000× *g*-force using an Optima L-100 XP Ultracentrifuge with a Type 45 Ti rotor (Beckman Coulter, CA, USA), and the TPNs were washed with DDIW in between the centrifugations. The purified TPNs were lyophilized and stored for future studies. The particle size, polydispersibility index (PDI), and zeta potential of TPN were characterized with 0.01 M SDS at pH 1.2 and pH 6.8 using a ZetaSizer Nano ZS (Malvern, Worcestershire, UK).

The morphology and size of dried TPN were examined using transmission electron microscopy (TEM). TPN was dispersed in either 0.1 N HCl (pH 1.2), pH 6.8 phosphate buffer solution, or DDIW using a probe sonicator for 15 min. Negatively charged carbon on copper grids (CF400-CU, EMS) were suspended with self-closing tweezers, and 4 microliters of sample were deposited onto the grid and left for 1 min. Excess liquid was removed by capillary force using wet filter paper. The sample was washed 3x with deionized water by pipetting 4 µL of water onto the grid and removing water by capillary forces using wet filter paper. Samples were then stained by depositing 4 µL of 2% uranyl acetate and left for 30 s before removing excess stain solution. Images were taken with a Talos L120C microscopy (ThermoFisher Scientific, MA, USA) using an accelerating voltage of 120 kV at 11,000× and 45,000× magnifications.

### 2.3. Preparation and Evaluation of Matrix Free-Films of VER-TPN-EC

#### 2.3.1. Film-Casting and Structural Characterization of Composite Matrix Formulations

To evaluate the influence of the TPN compositions listed in Table 1 on pH-dependent drug release, swelling, and microenvironmental pH, matrix free-films of TPN-EC or VER-TPN-EC were prepared by mixing 4 g of Surelease^®^ (1 g of EC), 0.1 g of VER (10% *w*/*w* based on EC weight of VER), and 0.1 g of TPN (10% *w*/*w* based on EC weight (pore former level) in 6 mL of DDI water, before stirring overnight. The TPNs were then dispersed using a probe sonicator for 15 min before being cast in a 6.35 cm diameter polytetrafluoroethylene plate and left to dry in a 40 °C oven for 24 h. The resultant free-film matrices were peeled off and stored at room temperature.

The chemical structures of the TPN, EC, and possible drug–polymer interactions involved in the dry powder mixture or films were examined using Fourier-transform infrared (FTIR) spectroscopy. FTIR spectra were obtained using an FTIR spectrometer (Spectrum 100, Perkin-Elmer, MA, USA) with a Universal ATR Sampling Accessory and a diamond coated zinc selenide (ZnSe) crystal plate (Perkin-Elmer, MA, USA). Samples were compressed against the crystal plate at low pressure by a micrometer clamp and dried under vacuum prior to measurement. Spectra were recorded with a resolution of 4 cm^−1^ and averaged over 32 scans. All spectra were normalized by the difference between the maximum and the minimum transmittance values.

#### 2.3.2. Determination of Drug Release from VER-TPN-EC Composite Matrix Films

Circular discs with 0.953 cm radius were cut from each of the drug-loaded TPN-EC matrix films made with TPN of different compositions (Table 1). For measuring drug release at pH 1.2, the discs were placed in 50 mL conical centrifuge tubes containing 50 mL of 0.1 N HCl for 24 h; for pH transition study, the discs were immersed in 37.5 mL of 0.1 N HCl for 2 h, followed by the addition of 12.5 mL of 0.2 M tribasic sodium phosphate to adjust the pH of the release media from 1.2 to 6.8. The tubes containing the samples were sealed with paraffin plastic film to minimize evaporation and were agitated via a horizontal shaker for 23 h. Release media were sampled using a closed loop peristaltic pump at predetermined time points, and drug concentration was assayed with a UV spectrophotometer (8453, Agilent, Waldbronn, Germany) at a wavelength of 278 nm.

Release rates were determined by taking the slope of drug release curve from 0–2 h at pH 1.2 and 3–8 h at pH 6.8 of dissolution. The degree of pH-dependence of release rates were evaluated by calculating the release rate difference between pH 1.2 and pH 6.8 as follows:

(1) $$Percent\;Release\;Rate\;Difference=\frac{pH\;1.2\;Release\;Rate-pH\;6.8\;Release\;Rate}{pH\;1.2\;Release\;Rate}$$

#### 2.3.3. pH-Dependence of Medium Uptake of TPN-EC Composite Matrix Films

Film specimens were cut with a circular punch die of 0.635 cm in diameter. The cut film specimens at dry state were weighed to obtain the initial weight (*w*_0_). The specimens were then placed in 0.1 N HCl or 6.8 buffer solution at 37 °C with horizontal shaking for 20 h. The samples were removed from the solution and blotted dry to remove the surface media before weighing to obtain the swollen weight (*w_s_*). The swollen samples were then dried at 50 °C for 20 h and reweighed to obtain the dry weight (*w_d_*). The %medium uptake (MU) was calculated as followed:

(2) $$MU=\frac{W_{s}-W_{d}}{W_{d}}\times 100\%$$

The relative ratio of %medium uptakes at pH 1.2 and pH 6.8 ($MU_{pH1.2}/MU_{pH6.8}$) was used to evaluate the pH-dependence of the medium uptake of the TPN-EC films.

#### 2.3.4. Preparation and Photography of pH Indicator Dye-Incorporated Matrix Films

To visualize the pH_m_ by color change, a pH indicator dye, bromocresol green, was incorporated into EC-based matrix films containing 10% TPN with mid-point composition (Table 1) (TPN-EC), or 10% PVP (PVP-EC). The films prepared as described in Section 2.3.1 were incubated in 0.1 N HCl for 2 h with agitation. The films were then removed and gently blotted dry and soaked in pH 6.8 phosphate buffer for an additional 22 h. Photographs of the films were taken using an Olympus E-M10 camera (Olympus Corporation, Japan), after removal from the media, and blotted dry. Each condition was performed in triplicates, and the most representative photographs were selected for the figures.

#### 2.3.5. Preparation and Measurement of pH_m_ of Fluorescent Dye-Labeled Matrix Films

A pH-sensitive fluorophore, SNARF-4F, was used to determine the pH_m_ of TPN-EC films with mid-point TPN composition exposed to external pH [36]. TPN-EC polymer dispersions with 10 mM of SNARF-4F were prepared as described in Section 2.3.1. Films were prepared in triplicates for each pH value by transferring 1 mL of polymer dispersion per sample to 96-well microplates and dried at 40 °C for 24 h. The finished films were incubated in media with external pH values of 1.2, 3, 5, and 7 for 2 h. Subsequently, the incubated films were imaged with a Xenogen IVIS Spectrum (Caliper Life Sciences Inc., Waltham, MA, USA) with two different filter channels. The excitation wavelength for each channel was 535 nm, while the emission intensities were measured at 580 nm (green channel) and 640 nm (red channel). The pH_m_ values of TPN-EC films were determined from the ratio of the intensity at 580 nm over 640 nm.

### 2.4. Preparation of TPN-Containing Bilayer-Coated Beads

Bilayer-coated beads with the inner drug-loaded matrix (VER-TPN-EC) and outer enteric coating membrane (TPN-EC) shown in Figure 1 were prepared by the two-step coating process described below.

#### 2.4.1. Application of Drug-Loaded Matrix Coating Layer (VER-TPN-EC)

Aqueous dispersions of VER, TPN, and Surelease^®^ (25% aqueous dispersion of EC) with various ratios of VER to TPN (i.e., 2:1, 1:1, and 1:2) (Table 2) were spray-coated onto MCC seed beads via a fluidized bed coater assembled with a bottom spray Wurster apparatus (ProCept 4M8 Fluid Bed, Zele, Belgium). The VER-TPN-EC dispersion was prepared by sonicating TPN in DDI water (Table 2) for 2 h with an ultrasonic processor (UP100H, Hielscher, Teltow, Germany) at 75% amplitude for 30 min, followed by the addition of Surelease^®^ and VER to produce 13% w/w total solid content (Table 2). The dispersion was stirred for 12 h and then sonicated with an ultrasonic processor at 75% amplitude for 30 min prior to coating. The MCC beads were coated using the following parameters: inlet temperature of 40–50 °C; air speed of 0.22 m^3^/min; nozzle size of 0.4 mm; air nozzle pressure of 1 bar; and spray rate of 1.5 g/min until a weight gain (WG) of 24% *w*/*w* was achieved, where % WG was calculated using the weight and solid content of the coating dispersion applied divided by the initial weight of the MCC beads loaded into the coating chamber.

#### 2.4.2. Application of TPN-EC Enteric Coating Membrane Layer

The ratio of TPN-EC in the membrane coating was fixed, according to Table 3. To prepare the dispersion for coating, TPN was first dispersed in DDI water and sonicated in the same manner as mentioned in the preparation of the drug-loading matrix dispersion. Next, Surelease^®^ was added to the dispersion to produce a solid content of 10% *w*/*w* (and stirred for 12 h, followed by 30 min of sonication. Application of the TPN-EC membrane onto the drug-loaded beads of three different VER:TPN ratios was performed with the same fluid bed setup and coating parameters as those used for the VER-TPN-EC layering process described above. During the coating process, bead samples were collected at five different coating levels: 5, 9, 13, 16, and 19% WG.

### 2.5. Determination of Drug Release from Bilayer-Coated Beads

Release of VER from the coated beads was determined using a modified USP dissolution apparatus II (VanKel VK7000, Varian Inc., Edison, NJ, USA) with 100 mL vessels and mini paddles, and a UV-Vis spectrophotometer (8453, Agilent, Waldbronn, Germany) configured with flow-through cuvettes to assay the drug concentration released into the bulk media. Beads equivalent to 12 mg of VER were placed in the vessels containing 90 mL of the dissolution medium at 37 °C and agitated at 200 rpm for 18 h. Dissolution tests were either conducted in media at a single consistent pH value (1.2 or 6.8) or in media that transitioned from pH 1.2 to 6.8. For the pH transition test, coated beads were first immersed in 67 mL of 0.1 N HCl. After 2 h of dissolution, the pH of the media was raised from 1.2 to 6.8 by adding 23 mL of 0.2 M tribasic sodium phosphate buffer, making the total volume 90 mL. Calculated total drug release was adjusted accordingly after 2 h with the increased media volume.

### 2.6. Statistical Analysis

One-way analysis of variance (ANOVA), Holm–Bonferroni method, and Student’s *t*-test were used to assess the statistical significance of the differences between formulations. A value of *p* < 0.05 was considered to be statistically significant, unless there were multiple comparisons. All the experiments and measurements were performed in triplicate (*n* = 3) with average ± standard deviation (SD), unless otherwise stated.

## 3. Results

### 3.1. Characterization of Synthesized TPN and TPN Films

#### 3.1.1. Particle Size, Zeta Potential, and Morphology of TPN

**Figure 2:**
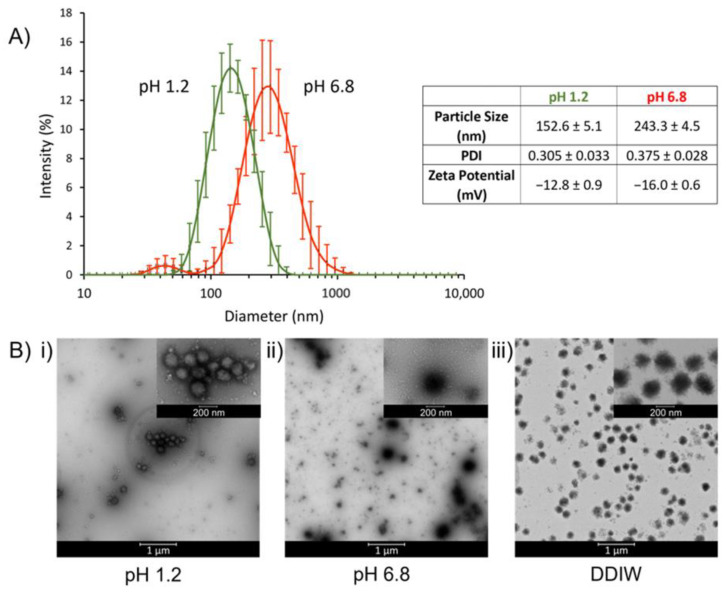
(**A**) Intensity-weighted hydrodynamic diameter and zeta potential of TPN of midpoint composition at pH 1.2 and pH 6.8. (**B**) TEM photographs of TPN with the midpoint composition dispersed in (i) 0.1 N HCl (pH 1.2), (ii) pH 6.8 phosphate buffer, or (iii) DDIW. The nanoparticles were stained with 2% uranyl acetate and dried on carbon-coated copper grids.

To investigate the particle size, size distribution, and zeta potential, TPN was dispersed at pH 1.2 and pH 6.8. At pH 1.2, TPN possessed a particle size of 152.6 ± 5.1 nm (PDI = 0.305) with a zeta potential of −12.8 ± 0.9 mV; and at pH 6.8, TPN had a particle size of 243.3 ± 4.5 nm (PDI = 0.375) with a zeta potential of −16.0 ± 0.6 mV (Figure 2A).

To examine the morphology and pH-dependent behavior of TPN, TEM photographs were taken at pH 1.2, pH 6.8, and DDIW, which showed TPN has a nearly spherical shape. It was observed that at pH 1.2, the size of TPN decreased to less than 200 nm and aggregated due to lack of ionic repulsion as they are unionized (Figure 2B(i)). At pH 6.8 and in DDIW, the edges of the TPN appear unsmooth, likely due to the extension of polymer chains, and minimal aggregation was seen due to ionic repulsion from ionization of the MAA (Figure 2B(ii,iii)). The particle size and zeta potential results correspond with the TEM results in various media, where TPN becomes more negatively charged in the presence of higher pH, leading to more electrostatic repulsion and therefore less aggregation (Figure 2B). These results conform with previous investigations of TPN, where the surface charge of the TPN is primarily contributed by the MAA [33,34].

#### 3.1.2. Investigation of Component Interactions within TPN-EC and VER-TPN-EC Films

**Figure 3:**
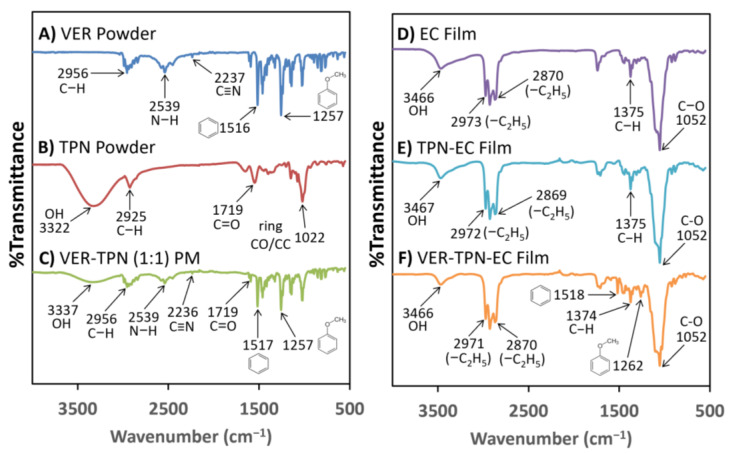
FTIR spectra of (**A**) pure VER powder, (**B**) pure TPN powder, (**C**) VER-TPN (1:1) powder mix (PM), (**D**) EC film, (**E**) TPN-EC film, and (**F**) VER-TPN (1:1)-EC film. Major peaks are denoted and further explained in the text.

FTIR spectra were analyzed to confirm the composition and possible interactions between EC, TPN, and VER in the TPN-EC and VER-TPN-EC films. Figure 3 exhibits infrared spectra of samples of pure VER powder, pure TPN powder, VER-TPN 1:1 powder mix (PM), pure EC film, TPN-EC film, and VER-TPN-EC films.

The FTIR spectrum of VER consists of major peaks at 2956 cm^−1^, 2539 cm^−1^, 2237 cm^−1^, 1516 cm^−1^, and 1257 cm^−1^, which are responsible for the stretching vibration of C-H, N-H, C ≡ N, the benzene ring, and C-O of the aromatic ethers of VER, respectively (Figure 3A). The spectrum of the TPN displays distinctive bands in the O-H and C-H regions, with a broad band around 3322 cm^−1^ and a strong peak at 2925 cm^−1^, respectively, which are consistent with previous findings (Figure 3B) [33]. The PM of VER and TPN at 1:1 ratio exhibits all major peaks from both VER and TPN, with the OH band at 3322 cm^−1^ shifting to 3337 cm^−1^ and a reduction in absorption (Figure 3C). The spectrum of EC film shows a broad band around 3466 cm^−1^, indicative of OH stretching, with 2870 cm^−1^ and 2973 cm^−1^, indicative of the -C_2_H_5_ stretching vibration peak; and the sharp peak at 1052 cm^−1^ suggests the presence of aliphatic ethers (Figure 3D). The spectrum peaks are all consistent with their known structure.

Upon the addition of TPN and VER to pure EC film, a sequential decrease and narrower band was observed at around 3466 cm^−1^ (Figure 3E,F), which is likely due to the dilution of TPN concentration within the films. In the VER-TPN-EC film, peaks from VER were observed at 1518 cm^−1^ and 1262 cm^−1^ (Figure 3F). These peaks were not seen in the TPN-EC and EC films, suggesting that VER maintains skeletal stretching of its benzene rings and stretching vibrations of its aromatic ethers. A juxtaposition of Figure 3A (VER) and Figure 3F (VER-TPN-EC) revealed the absence of the major peaks of VER at 2539 cm^−1^ and 2237 cm^−1^ responsible for the N-H stretching of the protonated amine, which suggests molecular interactions between VER and TPN via its N-H group with the carboxylic group of TPN (Figure 3F) [37].

### 3.2. Investigating the Effect of TPN Composition on Medium Uptake and Drug Release Using Free-Film Matrices

**Figure 4:**
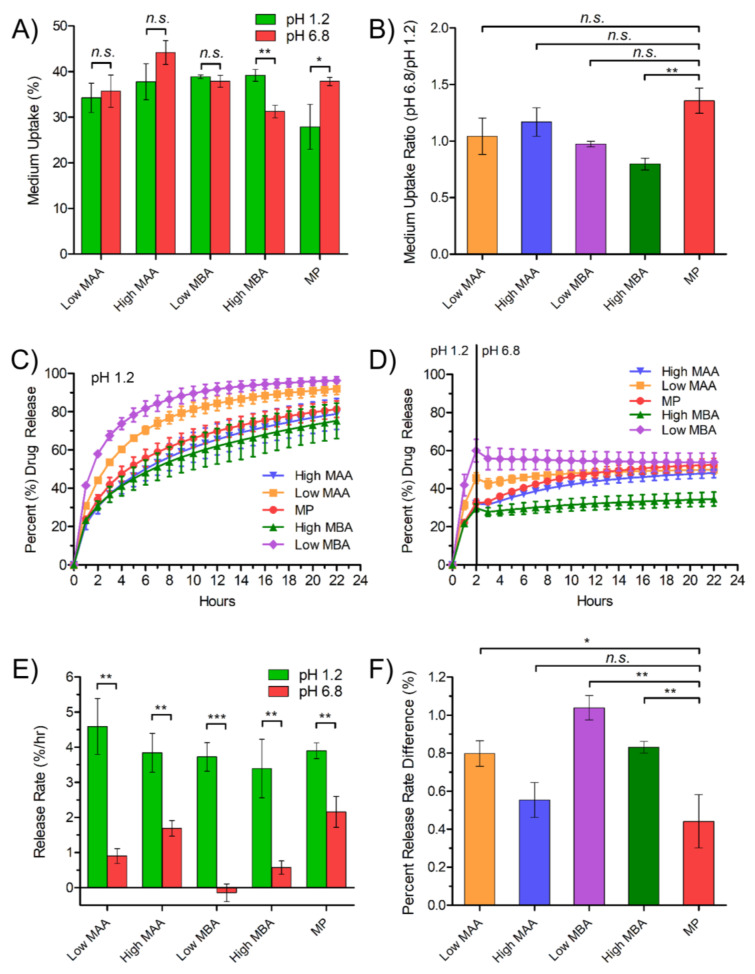
Effect of various TPN compositions on medium uptake and drug release rate. (**A**) Medium uptake from free-films at pH 1.2 or pH 6.8, (**B**) medium uptake ratio at pH 6.8 and 1.2, (**C**) drug release in pH 1.2 medium, (**D**) drug release in medium with pH transition from pH 1.2 to 6.8, (**E**) drug release rates from 0–2 h at pH 1.2 and 3–8 h at pH 6.8, and (**F**) percent release rate difference. The compositions of TPN denoted by Low MAA, High MAA, Low MBA, High MBA, and MP (midpoint) are listed in Table 1. Data are mean ± SD. *n.s.*, not significant, * *p* ≤ 0.05, ** *p* ≤ 0.01, and *** *p* ≤ 0.001. For multiple comparisons, *p* < 0.05/(*n*—rank + 1) was considered significant, where *n* is the number of comparisons.

To optimize the TPN composition, levels of MAA (ionizable moiety responsible for pH-dependent swelling and pH_m_) and MBA (cross-linker) were varied to yield a product capable of imparting greater permeability and local acidification at high pH to achieve a constant release rate during pH transition. The synthesized TPNs with the compositions listed on Table 1 fell within the particle size range consistent with previous studies [33,34]. The synthesized TPNs were freeze-dried and then incorporated into free-film matrices containing EC and VER with the compositions listed on Table 1. The medium uptake and drug release profiles of various TPN formulations were determined and are presented in Figure 4.

Among the TPN compositions evaluated, only MP demonstrated significantly higher medium uptake in pH 6.8 compared to pH 1.2, which is required for pH-responsive permeability (Figure 4A,B). Consequently, MP appeared to compensate most effectively for the reduced VER solubility at pH 6.8, as it exhibited the smallest change in release rate between pH 1.2 and 6.8 (Figure 4C–F). All TPN compositions exhibited first-ordered release pattern in pH 1.2, reaching a plateau around 20 h with approximately 64 to 98% of drug released (Figure 4C), but when tested in medium that transitioned from pH 1.2 to 6.8, the release rate of all compositions dropped immediately after the pH transition at 2 h, coinciding with the reduced VER solubility at higher pH (Figure 4D).

Following the pH transition, films with other compositions continued to release the drug at a lower rate, except for those with Low and high MBA, which failed to release drug further, despite exhibiting the fastest release at pH 1.2. The negative value of Low MBA release rate (Figure 4E) may indicate ionic complexation, or some degree of local precipitation following the pH change due to supersaturation. Because of a lower cross-linking density, Low MBA may undesirably uptake a high amount of medium, irrespective of pH (Figure 4A), thus making it harder to counteract the increase in pH_m_ from the high influx of medium after the pH transition. Additionally, any swelling effect of MAA at high pH would have been negligible within a matrix already low in density, which is shown by the insignificant difference in medium uptake (Figure 4A). High MBA, however, also exhibited a slower release rate in pH 6.8, correlated with the lowest medium uptake (Figure 4A,E). In this case, excess cross-linking density appeared to attenuate any swelling or pH_m_ effect MAA, and the insufficient diffusion passages within such a tight matrix impaired drug release over the entire duration (Figure 4D).

Comparing the levels of the acidic moiety, High MAA showed a high release rate in pH 6.8 (Figure 4E) and lower percent release rate difference (Figure 4F), similar to MP, though the difference in medium uptake was less significant between pH (Figure 4A). This suggested that a mechanism (i.e., supply of proton from MAA to lower pH_m_) other than medium uptake and retention may be responsible for the enhanced release.

In summary, the MP level, with moderate cross-linking and adequate MAA level, appeared to have the greatest advantage over all TPN compositions. MP exhibited the greatest ratio of media uptake at pH 6.8 compared to that at pH 1.2, which is suitable for pH-dependent swelling as an enteric coating agent (Figure 4B). At pH 6.8, the level of media uptake was similar to other compositions, while at pH 1.2 it was the lowest (Figure 4A). This composition was considered “optimized” among the various compositions studied. The balancing of MAA and MBA composition levels allowed for sufficient, though not excessive, media uptake at both pH 1.2 and 6.8, resulting in adequate lowering and maintenance of pH_m_. Although significant differences were found between the release rate values in pH 1.2 and 6.8 (Figure 4E), calculation of the percent release rate difference revealed the MP composition had the smallest change, where release rates at pH 1.2 compared to pH 6.8 were most similar, suggesting greater potential for pH-independent release with a substantial level of hydration and absorption of acidic media (Figure 4F).

### 3.3. Investigation of Microenvironmental pH within the Free-Film Matrices

To investigate the pH_m_ of the TPN-EC matrix, pH indicator dye, bromocresol green, and pH-sensitive fluorescent dye, SNARF-4F, were used. PVP-EC was used as a positive control within the pH indicator dye study because EC without pore formers is hydrophobic and will have minimal media uptake.

**Figure 5:**
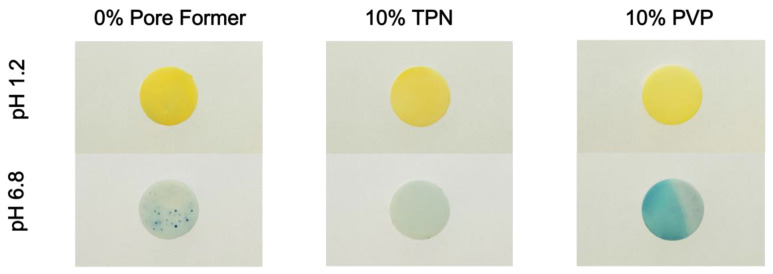
Photographs of EC, 10% TPN-EC, and 10% PVP-EC matrix films after incubation in pH 1.2 media for 2 h and then pH 6.8 for an additional 22 h containing bromocresol green.

In the presence of pH 1.2 media, all films appeared yellow, which was expected (Figure 5). After transition, PVP-EC showed a clear blue color, signifying that pH_m_ was higher than pH 5.4 (Figure 5). A similar result was seen in the EC film at areas where media uptake occurred. In contrast, TPN-EC film showed no strong blue color, but rather a greenish hue, Indicating the pH_m_ was between pH 3.8 and 5.4 (Figure 5). Overall, the results showed that the TPN-EC film maintained pH_m_ at minimal pH 5.4 for at least 22 h after transition.

**Figure 6:**
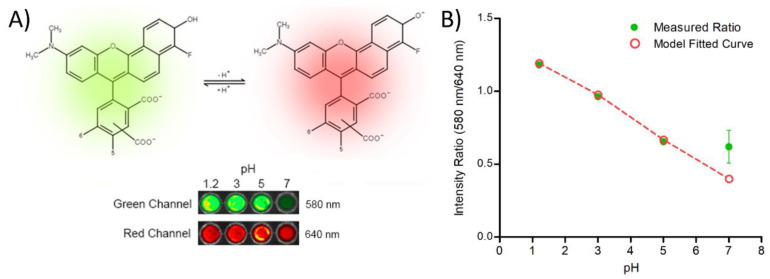
Measurement of pH_m_ using SNARF-4F with TPN at pH 1.2, 3, 5, and 7 for 2 h: (**A**) structural change of SNARF-4F and fluorescence imaging of SNARF-4F-loaded TPN-EC films and (**B**) intensity ratios of SNARF-4F with TPN at 580 nm and 640 nm. The dotted line represents the expected decrease in intensity ratio from pH 1.2 to 7 in absence of pH modulation provided by TPN.

The fluorescence imaging of SNARF-4F-loaded TPN-EC films further confirmed the effect of TPN on modifying the pH_m_ (Figure 6A). As the external pH value increased from 1.2 to 5, the intensity ratio of SNARF-4F fluorescence emission in the TPN-EC films decreased almost linearly; however, the measured result showed no statistical difference between pH 5 and pH 7 (Figure 6B). To predict the theoretical intensity ratio at pH 7 without pH_m_ modification by TPN, the measured data from pH 1.2 were fitted using the Boltzmann sigmoid equation (Equation (3)) [38]:

(3) $$pH=pK_{a}-log\left(\frac{R-R_{b}}{R_{a}-R}\times \frac{I_{b}}{I_{a}}\right)$$

where and *R* is the ratio of fluorescence intensity at individual pH, *R_a_* is measured ratio at acidic media, *R_b_* is measured ratio at alkaline media, and *I_a_* and *I_b_* are the fluorescent intensities at green and red channels, respectively; the *pK_a_* of SNARF-4F was set to 6.4, as per the supplier’s catalog [39].

The model fitted curve is presented in Figure 6B as a dashed line. It was found that the measured intensity ratio at pH 7 was ~60% higher than the expected intensity ratio as extrapolated from the fitted curve from pH 1.2 to 5 (Figure 6B). This result suggests that the pH_m_ of TPN-EC film is close to 5.3 when exposed to an external pH of 7, consistent with those obtained using the pH indicator dye (Figure 5).

### 3.4. Effect of VER:TPN Ratio in TPN-EC-Matrix Layer and TPN-EC Membrane Coating Level on Drug Release from TPN Bilayer-Coated Beads

**Figure 7:**
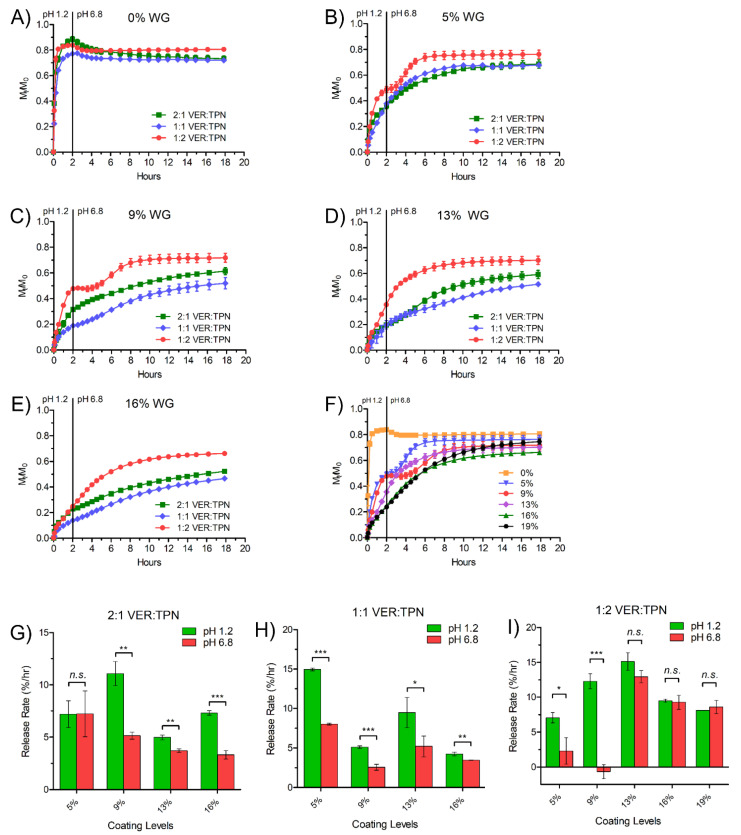
Dissolution profiles showing the effect of VER:TPN ratio in VER-TPN-EC matrix layer and TPN-EC membrane coating level. (**A**) 0%, (**B**) 5%, (**C**) 9%, (**D**) 13%, (**E**) 16% weight gain, and (**F**) 0% to 19% weight gain at 1:2 VER:TPN ratio, and comparison of VER release rates surrounding the pH transition at pH 1.2 (1–2 h) and pH 6.8 (2–3 h) between bilayer-coated bead formulations. (**G**) 2:1 VER:TPN, (**H**) 1:1 VER:TPN, and (**I**) 1:2 VER:TPN at various membrane coating levels. Data are mean ± SD. *n.s.*, not significant, * *p* ≤ 0.05, ** *p* ≤ 0.01, and *** *p* ≤ 0.001.

Because the experiments using VER-TPN-EC free-film matrices demonstrated the ability of TPN to modulate medium uptake and lower pH_m_, it was hypothesized that formulating the matrix into a bilayer-coated bead with an outer TPN-EC membrane coating could further modulate both the drug release out of the matrix and the influx of bulk medium into the matrix to sustain the pH_m_, achieving pH transition-independent controlled release. Using the optimized TPN composition (MP) determined above, beads were prepared to contain varying ratios of VER:TPN (2:1, 1:1, and 1:2) in the VER-TPN-EC matrix layer, and varying EC-TPN coating levels (0%, 5%, 13%, 16%, and 19% WG). The drug release profiles of these formulations were obtained under a pH transition condition (i.e., 2 h in pH 1.2 media follow by pH 6.8) and are presented in Figure 7A–F. From the release curves, drug release rates around the 2-h period (i.e., 1–2 h at pH 1.2 and 2–3 h at pH 6.8) were computed and are compared in Figure 7G–I. The insignificant difference in release rates between pH 1.2 and pH 6.8 is used as an indication of pH transition-independence of drug release rate.

As shown in Figure 7A, the beads coated with only the VER-TPN-EC matrix layer (0% WG) released most of the drug in the acid media, with a significant burst in the first 30 min, followed by a drop in apparent drug concentration after the pH transition, regardless of VER:TPN ratio. After application of the outer layer of TPN-EC, a range of controlled release profiles were obtained, all with reduced release rates at pH 1.2 while lifting the release curve at pH 6.8 with or without delay (Figure 7B–E). With the low coating levels (i.e., 5% and 9% WG) and a low VER:TPN ratio of 1:2, the beads exhibit a biphasic release profile (Figure 7B,C). At all coating levels, the beads with the highest TPN content (1:2, VER:TPN) produced the highest release rates at pH 1.2 and the highest overall release extent (Figure 7B–E). As the WG% increased to 13%, 16%, and 19%, the sharp transition disappeared, and the release profiles became a pseudo-zero order (Figure 7F).

VER release rates for all bilayer-coated bead formulations at pH 1.2 (1–2 h) and pH 6.8 (2–3 h) were computed and compared in Figure 7G–I. It can be seen that at pH 6.8, higher coating levels (13, 16, and 19% WG) of 1:2 VER:TPN ratio and lower coating level (5% WG) at 2:1 VER:TPN matched the release rates in pH 1.2, indicating pH-independent release (Figure 7G,I). This suggests that, for a given level of TPN in the matrix (which imparts hydrophilicity), there is an appropriate coating level required to regulate permeability and maintain the pH_m_ so that the release rate can be constant across the pH transition.

Although there appeared to be a trend of decreasing release extent with increasing coating level at 1:2, VER:TPN, the thickest coating (19% WG) offered the longest duration of zero-order release after pH transition, eventually exceeding the final extent of release of the preceding coating levels (except 5% WG, which failed to exhibit pH-independent release) (Figure 7F). This could be attributed to the greater uptake (of high TPN in the matrix) and retention (by high membrane coating level) of the acidic medium during the pH 1.2 phase of dissolution and thus maintaining the acidic pH_m_.

### 3.5. Achieving pH Transition-Independent Controlled Drug Release from Optimized TPN Bilayer-Coated Beads

**Figure 8:**
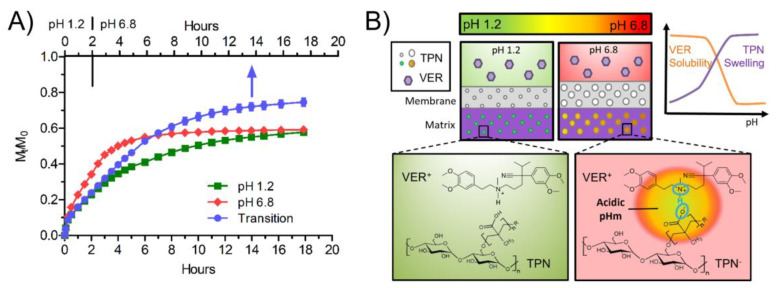
(**A**) Dissolution profiles of TPN bilayer-coated Beads at 19% weight gain, 1:2 VER:TPN ratio, in various pH conditions over full dissolution duration. (**B**) Proposed mechanism of pH_m_ and permeability modulation using TPN to achieve pH transition-independent controlled release from bilayer-coated beads. The combination of lowered pH_m_ generated within the inner matrix and increased permeability of the outer membrane at pH 6.8 enabled pH-independent release over a prolonged duration.

To further confirm our hypothesis of a medium uptake-based and pH_m_-modulating release mechanism of the bilayer-coated beads, full dissolution profiles (18 h) of the leading formulation (VER:TPN, 1:2 matrix with 19% WG membrane coating level) were conducted in pH 1.2 pH 6.8 medium, or with pH transition from pH 1.2 to 6.8 after two hours. The release profiles are compared in Figure 8A.

The initial release rate of VER in pH 6.8 only, compared to pH 1.2, was significantly higher (Figure 8A) despite the extreme difference in its solubilities, i.e., 6.5 mg/mL at pH 6.8 and >150 mg/mL at pH 1.2 This indicates that VER release is unlikely to be dictated by a single mechanism, as exhibited by conventional approaches used to merely attain a similar release rate at pH 6.8 compared to pH 1.2 using VER [9,40]. Instead, low membrane permeability at pH 1.2 was likely the governing factor for initial release, which also helped maintain acidic pH_m_ during the latter part of dissolution. The greater swelling of the coating due to ionization of TPN at a higher pH allowed a greater permeability and drug release rate, thereby overcompensating for the poor solubility of VER, whereas at lower pH, pores were relatively smaller, which lowers the drug release rate offsetting its higher solubility.

Compared to beads tested with a pH transition, beads tested in single pH media reached a lower extent of release. The earlier plateau of beads tested in pH 1.2 may be attributed to the reduced matrix and membrane permeability, whereas beads tested at pH 6.8 failed to release further due to the lower solubility of VER. It is plausible that some degree of local precipitation within the bead and/or ionic complexation between VER and ionized TPN played a role in slowing drug release after prolonged immersion in pH 6.8. The elevated release profile observed with the transition release profile might suggest that the initial uptake and later retention of acidic media are beneficial to prolong the pH_m_ effect of TPN, because it may provide an enriched source of protons to the MAA groups to help counter the exposure of pH 6.8 medial (Figure 8B). This result strongly reinforced the importance of both the pH-dependent swelling of TPN and microenvironmental modulation mechanisms, which are tailored to the drug solubility when designing a pH-independent release dosage form.

## 4. Discussion

In this work, we have demonstrated that the TPN, a crosslinked nanogel with weakly acidic component PMAA, when designed with an optimized composition (MP) and applied with EC in a bilayer bead formulation, is able to provide pH transition-independent controlled release of VER up to 18 h, despite the sharp drop in drug solubility from pH 1.2 to pH 6.8. The multifunctionality of TPN offers, as a single excipient, dual benefits as a pH_m_ modifier and an enteric coating agent in a bilayer bead formulation. In contrast, conventional formulations may rely on a combination of pH_m_ modifiers (e.g., fumaric acid) and enteric coating polymers (e.g., hydroxypropyl methylcellulose acetate succinate) to achieve release enhancement, which shows limited effectiveness due to their leachability and susceptibility to larger pores or cracks [40]. The non-leachable TPN has the unique advantage of creating nano-scaled pores in response to high pH for an extended time, which is especially important for maintaining the structural integrity of the dosage form for prolonged or extended release [41].

As illustrated in Figure 8B, the advanced performance of the TPN-containing bead can be ascribed to multiple functions of the TPN: (1) pH-dependent swelling that dictates the matrix or membrane permeability, counteracting the pH-dependent solubility of VER, i.e., lower permeability at pH 1.2 when VER solubility is high and higher permeability at pH 6.8 when VER solubility is low; (2) microenvironmental pH modification resulting in acidic pH_m_ in a composite matrix, which facilitates VER solution; and (3) forming pH-dependent ionic and ion-dipole complexation with VER, which retains VER in the nanogel, preventing its precipitation inside the matrix and the membrane. The mechanisms of the multifunctionality of TPN are further discussed below.

### 4.1. pH-Dependent Permeability Counteracts VER Solubility

Drug release from coated beads is dependent on its permeability through the membrane coating (Equation (4)).

(4) $$M=\frac{4\pi DKr_{o}r_{i}}{r_{o}-r_{i}}\;C_{d}t$$

where *D* is the diffusion coefficient (cm^2^/s), *K* is the partition coefficient, *r_o_* and *r_i_* are the outer and inner radii, respectively, of the membrane, *C_d_* is the saturated concentration of the drug in the reservoir, and *t* is time.

In our study, permeability, $\frac{DK}{r_{o}-r_{i}}$, was affected by coating level (i.e., thickness, *r_o_* and *r_i_*) and swelling of TPN, which impacted the effective *D* by changing the hydration degree, as described by Equation (5) [42,43]:

(5) $$\frac{D}{D_{w}}=Pexp\left(-\frac{Y}{Q-1}\right)$$

where *D* and *D_w_* are, respectively, the diffusion coefficient of drug in the TPN nanogel phase and in the medium; *P* is the sieving factor for the drug in the nanogel, related to the crosslinking density (e.g., the MBA content in the TPN); *Y* is a constant related to the characteristic solute volume and the free volume of water and *Y* = 1 for most polymer systems; and *Q* is the hydration/swelling degree, inversely proportional to the polymer volume fraction *φ*, *Q* = 1/*φ*.

Because EC is hydrophobic and essentially impermeable to the drug, pH-dependent TPN swelling dictates the permeability of the coating. At high pH, when MAA becomes ionized, electrostatic repulsion between polymer chains increases the sieving factor, *P*, and hydration degree, *Q*, leading to high permeability. However, it was highlighted that excess cross-linking could prevent adequate diffusion and impede drug release, therefore the MP level was ultimately selected for formulation into the bilayer beads because it demonstrated the most favorable balance between MAA and MBA among the various compositions studied.

Within the bilayer-coated beads, altering the ratio of VER to TPN in the matrix appeared to affect release extent. Although a high level of TPN relative to the drug appeared to enable more complete release due to higher matrix porosity, it was suspected that permeability was not the primary mechanism and that there was contribution by adjunct mechanisms (i.e., pH_m_). Instead, the pH-dependent permeability of TPN was effectively exploited by the outer membrane. By remaining relatively less permeable at low pH, the rapid release of VER (when its solubility is high) could be suppressed by minimizing contact between the bulk media and the drug, akin to enteric coating agents. Upon ionization and membrane swelling at higher pH, the drug could permeate at a higher rate to compensate for the low solubility of VER. To match the rates of release in both media, coating level was used to fine tune the permeability by altering the diffusion path length, where the highest level (19% WG) with high TPN (1:2, VER, TPN) was most effective.

### 4.2. Microenvironmental pH Modification by TPN

In addition to enhanced permeability at high pH, acidification of pH_m_ by TPN was likely a contributor to pH-independent release of VER from the bilayer-coated bead. TPN was able to lower the pH_m_ of TPN-EC films by a value of approximately 2 pH from pH 7 of the bulk medium (Figure 6), corresponding to an increase in VER solubility of over 50-fold [9]. The observed increase in drug release from TPN-EC films and coated beads in the pH 6.8 buffer further suggests the effect of TPN on modulating the pH_m_.

The ability of the TPN-EC film matrix to maintain its acidic pH_m_ in the presence of pH 6.8 buffered media could be explained by Donnan equilibrium between the polymer matrix/membrane and the bulk medium. The nanogel TPN-containing ionic film can generate an uneven distribution of charged species across the internal and external sides [44,45,46]. On the one hand, the ionization of PMAA in the TPN-EC film matrix, i.e., acquiring a negative charge, at higher pH may have reduced the diffusion and accumulation of anionic phosphate buffer ions into the film matrix due to electrostatic repulsion, resulting in less phosphate buffer ions inside the film to neutralize the acidic microenvironment. On the other hand, the unleachable anionic PMAA-containing TPN attract substantially higher counterions, i.e., protons, to achieve an electrostatic neutralization. This phenomenon can be described as follows:

(6) $$C_{A}^{\prime }=\lambda^{z_{i}}C_{A}$$

where $C_{A}^{\prime }$ and $C_{A}$ are the concentration of counter ions in the film matrix and in the bulk solutions, respectively; λ is the Donnan ratio, which relates the concentrations of anionic ions in the film matrix to the concentrations in the bulk solution; and $z_{i}$ is the valence of the corresponding *i*th ionic species. For monobasic and dibasic phosphate buffer species, $z_{i}$ is equal to 1 and 2, respectively.

λ can be calculated using the charge balance of the solution and the matrix film as follows:

(7) $$\left(1-\varphi \right)\sum z_{i}\lambda^{z_{i}}C_{i}-\frac{\sigma \varphi }{1+\lambda 10^{{\mathrm{pK}}_{a}^{\prime }-\mathrm{pH}}}=0$$

where *φ* and σ are the polymer volume fraction of the ionized TPN and the molar density of charged groups, respectively; ${\mathrm{pK}}_{a}^{\prime }$ is the acid dissociation constant of the PMAA groups of TPN; and pH is the pH of the external solution. The value of λ is given by the single real positive root of Equation (7).

Taking the negative logarithm of Equation (6), the pH_m_ value relative to the external pH is then given by applying Equation (6) to free protons:

(8) $${\mathrm{pH}}_{m}=\mathrm{pH}-\log\lambda$$

Based on the highest estimate of pH_m_ ~ 5.4, determined from the pH indicator and fluorescence imaging (Figure 5 and Figure 6), applying external pH = 6.8, the λ is estimated to be 25. In other words, the proton concentration in the films is about 25-fold that in the medium.

The Debye–Hückel theory, in conjunction with the Donnan equilibrium, may also be used to explain the pH-independent release of the bilayer-coated beads [47], which was most effective when using high TPN content (1:2, VER:TPN) with high coating level (19% WG). In the presence of pH 6.8 phosphate buffer solution, the MAA groups of the outer TPN-EC membrane coating layer were first ionized. As the ionization degree of the membrane layer increases, more Na^+^ ions diffuse in. Because Na^+^ ions have a lower binding affinity than H^+^ ions to the MAA unit, more protons were retained in the TPN hydrogel, suppressing further ionization of the MAA groups of the inner matrix layer [48,49]. The hindered entry of anionic phosphate ions and preferred retention of protons lead to a more acidic pH_m_ of the inner matrix, relative to the bulk pH. Thereby, the bilayer-coated beads could lower the pH_m_ of the inner matrix layer to a greater degree than a single matrix layer could achieve. Hence, pH-independent release was demonstrated with bilayer-coated beads, but not with single-layer film matrices. Higher charge density would result in greater electrostatic repulsion and in turn more acidic pH_m_, as was seen with TPN containing higher MAA content showing more pH-independent release (Figure 4A,B).

### 4.3. pH-Dependent Complexation

Incomplete drug release is common for weakly basic drugs with pH-dependent solubility, because the driving force for release decreases as pH increases and as the drug in the reservoir is depleted. This phenomenon is especially pronounced in controlled release formulations, where a barrier is present, because exposure of the drug to the media is decreased [50]. The concentration of VER in the media is well below sink conditions and so incomplete release cannot be attributed to drug precipitation within the media. However, some local supersaturation within the matrix or membrane pores may have led to internal precipitation, halting further drug release. Alternatively, 25% of the remaining drug within the beads in our study may be attributed to ionic complexation or ion dipole interaction of VER with the negatively charged TPN, in addition to the Donnan equilibrium [32]. While this may seem like a disadvantage, the complexation or ion dipole interactions allow VER to remain within the inner matrix longer in a more acidic pH_m_, allowing VER to remain longer within the drug reservoir and preventing drug–drug interaction (i.e., precipitation) when exposed to high pH. This was seen from the results that formulations containing greater TPN content exhibited longevity of upward release extent. Further optimization, such as finer tuning of drug load and TPN levels, may yield greater overall release extents.

## 5. Conclusions

In this work, the multifunctionality of a nanogel TPN was investigated as a non-leachable pH_m_ modifier and pH-responsive pore former in a bilayer-coated bead formulation for the pH transition-independent controlled release of weakly basic drugs. The results demonstrated that incorporation of TPN, comprised of pH-sensitive MAA and cross-linker MBA, enables the inner matrix layer of VER-TPN-EC to maintain a pH_m_ approximately 1.5 unit lower than the external buffer pH, which may be further enhanced by coating with a TPN-EC membrane. In a simulated gastric and intestinal pH transition condition (i.e., from pH 1.2 to 6.8), the bilayer-coated beads with 16% to 19% WG resulted in a constant release rate during the pH transition, followed by a sustained release of VER up to 18 h, beyond the extent achieved when tested in single pH media. This ability to overcome the poor solubility of VER at high pH can be ascribed to the combinatory effects of the pH-dependent swelling of TPN that increased permeability, preferred retention of protons in the TPN due to Donnan equilibrium, pH-dependent complexation between MAA and VER, and the barrier to diffusion of buffer ions by the outer coating. This work suggests that the multifunctionality and tunability of TPN-EC formulations and the formulation design strategy may be expanded to tackle the challenges faced by other drugs with severe pH-dependence of water solubility.

## Figures and Tables

**Table 1:** Feed levels of the MAA, MBA, PS 80, and starch used during synthesis of TPNs with various compositions.

TPN Composition	Variables (mmol)		MAA:MBA:PS 80:Starch Molar Ratio			
	MAA (x_1_)	MBA (x_2_)	MAA	MBA	PS80	Starch
Low MAA	13.47	3.24	2.75	0.66	0.12	1.00
High MAA	33.01	3.24	6.74	0.66	0.12	1.00
Mid-point (MP)	23.24	3.24	4.74	0.66	0.12	1.00
Low MBA	23.24	0.52	4.74	0.11	0.12	1.00
High MBA	23.24	5.97	4.74	1.22	0.12	1.00

**Table 2:** Composition of VER-TPN-EC dispersion for making matrix films.

Materials	VER:TPN (%*w*/*w* of Dispersion)		
	2:1	1:1	1:2
VER	2	2	2
TPN	1	2	4
Surelease^®^	40	36	28
DDI water	57	60	66

**Table 3:** Composition of TPN-EC membrane coating dispersion.

Materials	%*w*/*w* of Dispersion	%*w*/*w* of Solid Content
TPN	1	10
Surelease^®^	36	90
DDI water	63	–

## Data Availability

The data presented in this study are available in this article.
